# The Impact of Helicobacter pylori on Marginal Ulcer Formation Following Gastric Bypass: A Systematic Review and Meta-Analysis

**DOI:** 10.7759/cureus.96787

**Published:** 2025-11-13

**Authors:** Arifur Rahman, Muhammad Mushtaq, Faisal Nadeem, Syed A Kabir

**Affiliations:** 1 General Surgery, Walsall Manor Hospital, Walsall, GBR; 2 General and Bariatric Surgery, Walsall Manor Hospital, Walsall, GBR

**Keywords:** gastric bypass surgery, h.pylori infection, marginal ulcer, roux-en-y gastric bypass (rygb), systematic review and meta-analysis

## Abstract

Roux-en-Y gastric bypass (RYGB) is one of the most commonly performed bariatric procedures worldwide and is highly effective in improving obesity-related comorbidities; however, marginal ulceration remains a recognised postoperative complication. *Helicobacter pylori* (HP), a known risk factor for peptic ulcer disease, has an uncertain role in marginal ulcer formation following RYGB. This systematic review and meta-analysis aimed to clarify whether preoperative HP infection increases the risk of marginal ulcer development. A systematic search of PubMed, Embase, and Cochrane databases identified 119 studies, of which seven met inclusion criteria, encompassing 255,899 patients (mean age 42.8 years, mean BMI 47.3 kg/m²). Pooled analysis using a random-effects model demonstrated no significant association between HP infection and marginal ulcer incidence after RYGB (risk ratio = 1.16, 95 % CI 0.23-5.88, p = 0.86), with substantial heterogeneity among studies (I² = 96.4 %, p < 0.0001). These findings suggest that preoperative HP infection does not significantly influence the risk of marginal ulcer formation following RYGB.

## Introduction and background

Obesity is a growing global epidemic, with an estimated 25% of the world’s population projected to be obese by 2035 [1]. The burden on healthcare systems is substantial, with obesity-related conditions costing the UK National Health Service approximately £11.4 billion annually, alongside an additional £74.3 billion loss to the wider economy due to reduced productivity [2]. For patients with morbid obesity who have failed conservative management, bariatric surgery remains the most effective intervention for sustained weight loss and metabolic improvement [3]. Among the available procedures, the Roux-en-Y gastric bypass (RYGB) is one of the most commonly performed worldwide, with approximately 2,500 procedures undertaken annually in the UK [4].

Despite its efficacy, RYGB carries several postoperative complications, one of the most clinically significant being marginal ulceration (MU) [5]. Marginal ulcers are mucosal defects that typically occur at the gastrojejunal anastomosis and are believed to result from multiple interacting factors including gastric acid exposure, surgical technique, smoking, non-steroidal anti-inflammatory drugs (NSAIDs) use, and bacterial infection [6]. Helicobacter pylori (H. pylori; HP) is a well-established cause of peptic ulcer disease, as it is known to induce mucosal inflammation and acid hypersecretion. This has been proposed as a potential risk factor for MU formation as it could theoretically impair healing at the surgical junction [7].

Although multiple studies have examined the relationship between H. pylori and MU after gastric bypass, most have assessed it as one of several variables rather than the primary focus of investigation [8]. Furthermore, the findings have been inconsistent, with some reporting a positive association while others found no significant link [9,10]. Given the increasing prevalence of both obesity and H. pylori infection, understanding this relationship is essential for optimising perioperative management and prevention strategies. This systematic review and meta-analysis aims to synthesise the existing evidence on the impact of H. pylori infection on marginal ulcer formation following gastric bypass surgery.

## Review

Methods

This systematic review and meta-analysis was conducted in accordance with the Preferred Reporting Items for Systematic Reviews and Meta-Analyses (PRISMA) 2020 guidelines. A comprehensive search was performed using PubMed, Embase, and the Cochrane Library to identify relevant studies evaluating the association between Helicobacter pylori infection and marginal ulcer formation following gastric bypass surgery. Only studies published in English and involving human participants were included.

The search strategy incorporated combinations of controlled vocabulary and free-text terms including: “Roux-en-Y gastric bypass,” “gastric bypass,” “RYGB,” “RNYGB,” “bariatric surgery,” “marginal ulcer,” “anastomotic ulcer,” “gastrojejunal ulcer,” “stomal ulcer,” “jejunal ulcer,” “peptic ulcer,” “Helicobacter pylori,” “H. pylori,” and “Helicobacter infections.” The PubMed and Embase (Ovid) search strings were constructed using Boolean operators and adapted for each database’s indexing system. The full search strings used can be found in the appendix. The final search was performed on 20/09/25.

Studies were eligible if they included adult human subjects undergoing primary Roux-en-Y gastric bypass and reported outcomes relating to H. pylori infection and marginal ulcer formation. Case reports, review articles, animal studies, non-English publications, studies of revisional gastric bypass, and those with insufficient outcome data were excluded.

Two reviewers independently screened titles and abstracts using the Rayyan platform. Full texts were retrieved for potentially relevant studies, and discrepancies were resolved by discussion or arbitration by a third reviewer. Data were extracted independently by two reviewers, including study design, year of publication, country, sample size, diagnostic method and timing of H. pylori testing, prevalence of infection, incidence of marginal ulceration, and reported associations between H. pylori and ulcer outcomes.

The risk of bias of all included studies was evaluated independently by two reviewers using the Newcastle-Ottawa Scale (NOS) for cohort studies. All studies met acceptable methodological standards, with the majority scoring between five and nine points, indicating moderate to high quality. No studies were classified as high risk of bias according to NOS criteria.

All data were handled in accordance with PRISMA guidelines, with independent verification of extracted information and no imputation required due to complete reporting across included studies.

Statistical analysis for the meta-analysis was conducted using Review Manager employing a random-effects model.

Ethical approval was not required, as this study involved secondary analysis of published data.

Results

Study Selection

**Figure 1:**
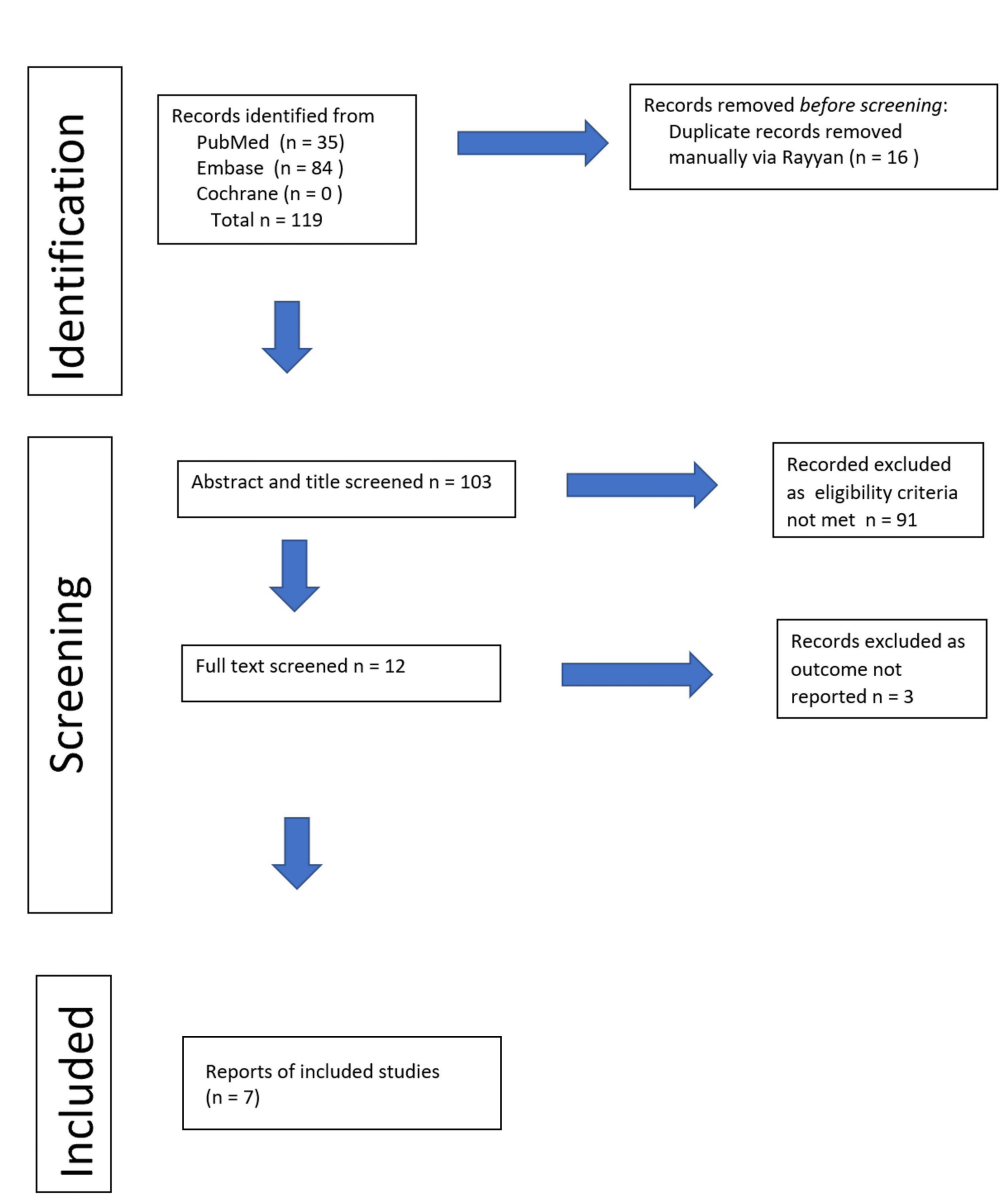
Study identification and screening algorithm as per Preferred Reporting Items for Systematic Reviews and Meta-Analyses (PRISMA) 2020

The initial database search of PubMed, Embase, and Cochrane identified 119 records. After removal of 16 duplicates, 91 articles were excluded following title and abstract screening for not meeting inclusion criteria. A further three full-text papers were excluded for non-relevant outcomes. Ultimately, seven studies met the inclusion criteria and were included in the quantitative synthesis (Figure 1).

Study Characteristics

All seven included studies were retrospective observational designs, comprising a total of 255,899 patients. Six studies were conducted in the United States and one in Taiwan. Table 1 summarises patient demographic data. The mean age across studies was 42.8 years, with a mean BMI of 47.3 kg/m²; 16% of participants were male, reflecting a predominantly female bariatric population. 

**Table 1:** Patient Characteristics from Selected Studies

Author (Year)	Mean Age (yrs)	Mean BMI (kg/m²)	Gender (M/F%)
Rasmussen et al. (2007) [9]	42	44	10 / 90
Kelly et al. (2015) [10]	46	47	24 / 76
Rawlins et al. (2013) [11]	46.5	45	24 / 76
Schulman et al. (2017) [12]	44	–	19 / 81
Yang (2006) [13]	35	41.6	–
Loewen et al. (2008) [14]	40.6	48.6	13 / 87
Papasavas et al. (2008) [15]	45.1	46	19 / 81
Pooled mean (± SD*)	42.8	47.3	16% male (84% female)

Methodological details are presented in Table 2. Most studies performed pre-operative HP testing using serology or gastric biopsy; one large registry study utilised administrative International Classification of Diseases, Ninth Revision (ICD-9) coding. HP eradication was confirmed in three studies, partially reported in two, and not mentioned in the remainder. Follow-up durations ranged from 10.2 to 24 months. MU were defined as endoscopically confirmed ulcers in symptomatic patients in six studies, and by ICD-9 coding in one large database cohort.

**Table 2:** Study Characteristics ICD-9: International Classification of Diseases, Ninth Revision, ELISA: enzyme-linked immunosorbent assay, EGD: esophagogastroduodenoscopy

Author (Year)	Country	Study Design	Pre-operative H. pylori Testing
Rasmussen et al. (2007) [9]	USA	Retrospective	Pre-operative ELISA serology
Kelly et al. (2015) [10]	USA	Retrospective	Intra-operative full-thickness gastric biopsy (Warthin–Starry stain)
Rawlins et al. (2013) [11]	USA	Retrospective cohort	Pre-operative ELISA serology + endoscopic biopsy
Schulman et al. (2017) [12]	USA	Retrospective database cohort	Administrative ICD-9 coding for H. pylori
Yang (2006) [13]	Taiwan	Retrospective cohort	Pre-operative ELISA serology
Loewen et al. (2008) [14]	USA	Retrospective	Pre-operative gastric biopsy during routine EGD
Papasavas et al. (2008) [15]	USA	Retrospective	Pre-operative ELISA serology

Quantitative Analysis

Outcomes of MU incidence in HP-positive and HP-negative cohorts are summarised in Table 3. Across the pooled dataset, 607 HP-positive and 255,131 HP-negative patients were analysed.

**Table 3:** Marginal Ulceration (MU) Incidence in Presence or Absence of H. pylori (HP)

Author (Year)	HP⁺ with MU (N)	HP⁺ without MU (N)	Total HP⁺ (N)
Rasmussen et al. (2007) [9]	6	29	35
Kelly et al. (2015) [10]	5	61	66
Rawlins et al. (2013) [11]	1	67	68
Schulman et al. (2017) [12]	107	234	341
Yang (2006) [13]	6	26	32
Loewen et al. (2008) [14]	1	6	7
Papasavas et al. (2008) [15]	2	56	58

**Figure 2:**
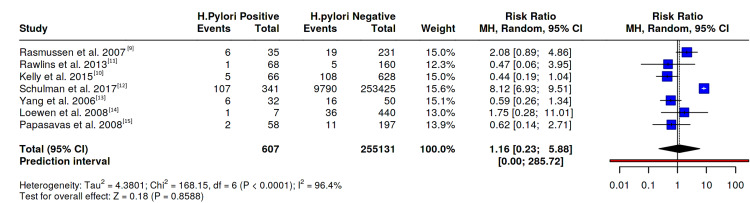
Meta-analysis of marginal ulceration (MU) incidence in H. pylori positive and negative patients

The forest plot (Figure 2) summarises the individual and pooled risk ratios comparing pre-operative Helicobacter pylori-positive and -negative patients for marginal ulcer formation after Roux-en-Y gastric bypass. Individual study estimates varied widely in both direction and magnitude. Five studies suggested no significant association, with confidence intervals crossing the line of no effect (risk ratio (RR) = 1), while the large database study by Schulman et al. (2017) showed a markedly increased risk and contributed the highest weighting (15.6%) to the pooled estimate. The remaining studies carried relatively balanced weights (12-15%), reflecting comparable sample sizes.

A random-effects meta-analysis yielded a pooled RR of 1.16 (95% CI 0.23-5.88), indicating no statistically significant association between pre-operative Helicobacter pylori infection and the development of marginal ulcers following RYGB (p = 0.86). Heterogeneity was considerable (I² = 96.4%, p < 0.0001). The corresponding 95% prediction interval (0.00-285.72) was extremely wide, implying that the true effect in future comparable studies could range from a strong protective effect to a markedly increased risk. This broad interval reflects substantial uncertainty, likely arising from differences in study design, diagnostic criteria, and patient selection across included studies. Sensitivity analysis excluding the large database study by Schulman et al. (2017) produced a similar pooled estimate, confirming the robustness of the overall findings [12].

Discussion

Marginal ulceration remains one of the most frequent late complications following RYGB, with multifactorial aetiology including surgical technique, smoking, NSAID use, and Helicobacter pylori infection. This systematic review is the first to evaluate specifically the association between H. pylori and marginal ulcer formation following gastric bypass surgery. Seven studies formed the evidence base for this analysis [9-15]. Collectively, no statistically significant relationship was observed, although the direction of effect in several studies suggested a potential trend.

Rasmussen et al. (2007) were among the first to identify a possible link between preoperative H. pylori infection and marginal ulceration, findings later supported by Schulman et al. (2017) in a large database study of over 250,000 patients [9,12]. However, these studies relied on retrospective data and, in the case of Schulman et al., administrative coding rather than clinical confirmation [12]. By contrast, Yang et al. (2006) [13], Rawlins et al. (2013) [11], and Kelly et al. (2015) [10] found no association between H. pylori and MU risk. A systematic review by Beran et al. (2022) suggested a possible relationship but included only three relevant studies and did not analyse H. pylori as the primary exposure [8].

The strength of this review lies in its focused scope, comprehensive search, and synthesis of both institutional and national datasets. Nevertheless, several limitations constrain interpretation. All included studies were retrospective, and five were based in the United States, where H. pylori prevalence is relatively low [16]. Diagnostic heterogeneity in both H. pylori testing (serology, biopsy, ICD codes) and MU definition (endoscopic vs coding-based) contributes to variability [8]. Additionally, potential confounders such as smoking, NSAID use, and pouch size were inconsistently reported [8].

While the findings do not support a statistically robust link between H. pylori and marginal ulceration, routine preoperative eradication may still be reasonable, particularly in high-prevalence regions or symptomatic patients. Future research should focus on prospective, multicentre studies with standardised diagnostic protocols, uniform definitions of MU, and adequate control of confounding variables.

## Conclusions

Overall, current evidence does not demonstrate a statistically significant association between H. pylori infection and marginal ulcer formation after gastric bypass surgery. Larger, standardised prospective studies are required to determine whether preoperative H. pylori eradication meaningfully reduces marginal ulcer risk.

## Appendices

Search strategy

Date of last search: 20 September 2025 Databases searched: PubMed (MEDLINE)

(("Roux-en-Y gastric bypass"[MeSH Terms] OR "Roux-en-Y gastric bypass"[tiab] OR "gastric bypass"[tiab] OR "RYGB"[tiab] OR "RNYGB"[tiab] OR "bariatric surgery"[tiab]) AND ("marginal ulcer"[MeSH Terms] OR "marginal ulcer"[tiab] OR "anastomotic ulcer"[tiab] OR "gastrojejunal ulcer"[tiab] OR "stomal ulcer"[tiab] OR "jejunal ulcer"[tiab] OR "peptic ulcer"[tiab]) AND ("Helicobacter pylori"[MeSH Terms] OR "Helicobacter pylori"[tiab] OR "H. pylori"[tiab] OR "Helicobacter infection*"[tiab])) NOT ("animal"[MeSH Terms] NOT "human"[MeSH Terms])
 

Databases searched: Embase (Ovid)

('roux en y gastric bypass'/exp OR 'roux-en-y gastric bypass':ti,ab OR 'gastric bypass':ti,ab OR 'rygb':ti,ab OR 'rnygb':ti,ab OR 'bariatric surgery':ti,ab) AND ('marginal ulcer'/exp OR 'marginal ulcer':ti,ab OR 'anastomotic ulcer':ti,ab OR 'gastrojejunal ulcer':ti,ab OR 'stomal ulcer':ti,ab OR 'jejunal ulcer':ti,ab OR 'peptic ulcer':ti,ab) AND ('helicobacter pylori'/exp OR 'helicobacter pylori':ti,ab OR 'h. pylori':ti,ab OR 'helicobacter infection*':ti,ab) NOT ([animals]/lim NOT [humans]/lim) AND ([english]/lim)
